# Management and Outcomes of Anorectal Malformations at University Teaching Hospital of Kigali, Rwanda from 2015 to 2023

**DOI:** 10.21203/rs.3.rs-7681835/v1

**Published:** 2025-09-24

**Authors:** Chioma Moneme, Owen Selden, Kimberley Duru, Shaina Twardus, Jordan Gooding, Jean Pierre Habumufasha, Aimable Kanyamuhunga, Ruyun Jin, Tracy Kelly, Sandra Kabagambe, Edmond Ntaganda

**Affiliations:** University of Virginia; University of Virginia; University of Virginia; University of Virginia; University of Virginia; University Teaching Hospital of Kigali; University Teaching Hospital of Kigali; University of Virginia; University of Virginia; University of Illinois Urbana-Champaign; University Teaching Hospital of Kigali

## Abstract

**Purpose:**

ARMs are common congenital disorders, including LMICs, where limited neonatal and surgical care leads to worse outcomes compared to HICs. Recent efforts to improve health systems in LMICs focus on training healthcare workers. This study assesses the clinical care of infants with ARMs at the University Teaching Hospital of Kigali (CHUK), a tertiary-level hospital in Rwanda, provided by pediatric surgeons and other fellowship-trained pediatric specialists.

**Methods:**

A retrospective study of patients who presented with ARMs at CHUK, Rwanda, from January 2015 to December 2023. All patients who underwent their definitive anorectoplasty at the CHUK and had retrievable data in operative logbooks were included. Regression analysis was performed to determine predictors of 30-day mortality.

**Results:**

62 patients were included. The most common fistula type was a rectovestibular fistula (48%) in females and a rectoperineal fistula (18.9%) in males. 43.5% underwent a three-stage repair. 30-day mortality was 17.9%. Low weight (<3000g) at surgery was significantly associated with 94% higher odds of mortality.

**Conclusion:**

Although mortality was higher than that encountered in HICs, it was lower than what has generally been reported in LMICs. This could be attributed to the improved care provided by fellowship-trained pediatric specialists.

## Introduction

Anorectal malformations (ARM) constitute a broad spectrum of congenital disorders resulting in abnormal development of the rectum, anus, and urogenital structures.^1^ ARM is present in 1 out of every 5,000 live births and has a greater prevalence in males than in females at a 1.2:1 ratio.^2^. The underlying etiology of ARM remains poorly understood. Specific factors consistently associated with anorectal malformations include multiple gestation, preterm birth, maternal obesity, and diabetes (Wijers et al). ^78,9^ Apart from occurring in isolation, anorectal malformations have an association with other congenital malformations, including trisomy 21, VACTERL (Vertebral, Anal, Cardiac, Tracheo-Esophageal, Renal and Limb) association, and caudal regression syndrome.^9,10^

According to the World Health Organization (WHO), an estimated 6% of infants are born with a congenital disorder each year worldwide, with approximately 94% of those infants born in low- and middle-income countries (LMICs). There is also a larger proportion of deaths caused by congenital malformations in this population.^11^ An extensive international prospective study showed similar representation of esophageal atresia and Hirschsprung disease in high-income countries (HICs) and low-to middle-income countries (LMICs). However, the same study also showed a higher presentation of anorectal malformations in LMICs compared to HICs.^12^ Advances in prenatal screening have contributed to increased detection of congenital anomalies in utero. However, diagnosis of ARM in the prenatal period remains challenging, resulting in the majority of cases being diagnosed in the early neonatal period instead.^9^ The diagnosis of ARMs is most commonly classified using the Krickenbeck consensus classification system, which also guides management.^13^

Furthermore, limited prenatal screening programs, limited resources, and a lack of trained providers contribute to widening disparities in the management of congenital malformations.^14^ Surgical intervention is the definitive treatment for anorectal malformations, but limited capacity in LMICs due to a significant shortage of pediatric surgeons, pediatric anesthesiologists, and trained personnel, amongst other factors, poses a challenge in LMICs where nearly 50% of the population is under 15 years of age.^15,16^ Consequently, while mortality rates are as low as 3% in HICs, mortality rates are as high as 20% in LMICs^17–19^. This disparity in mortality has been linked with delays in diagnosis and treatment, as several factors, including geographic distance to referral hospitals, are associated with limited healthcare access.^20^

There are few studies describing pediatric outcomes of congenital malformations in LMICs that have made strides towards improving the quality of pediatric surgical care. Rwanda is a low-income country, with 40% of its population under 15 years of age. Rwanda operates a tiered health system including community health centers, primary health centers, district hospitals, and referral hospitals, the latter providing the highest level of specialized care. Before early 2010, referral hospitals in Rwanda were relying on general surgeons and visiting international pediatric surgeons for operative management of congenital gastrointestinal anomalies.

Surgical intervention for anorectal malformations has been previously described in literature in a retrospective review of forty-six cases performed between 2002 and 2007 at the University Teaching Hospital of Butare, Rwanda. However, there have been no further studies in the literature since the introduction of a fellowship-trained pediatric surgeon and fellowship training program. In this study, we aim to assess the overall clinical landscape, including clinical factors associated with increased mortality, of infants with anorectal malformations at a referral hospital in Rwanda under the care of fellowship-trained pediatric specialists, including pediatric surgeons and neonatologists.

## Methods

We conducted a retrospective cohort study of infants evaluated and treated for congenital ARMs at Centre Hospitalier Universitaire de Kigali (CHUK) from January 2015 to December 2023. CHUK is a large referral and teaching hospital located in Kigali, Rwanda, that serves patients from all over the country with a specific catchment area covering almost 70% of the country for pediatric surgical cases.. Patients who presented at CHUK with an ARM diagnosis without a prior history of anorectoplasty were eligible for inclusion. Patients who had undergone colostomy prior to presenting at CHUK were eligible for inclusion. Eligible patients were identified from operating theatre logbooks and pediatric ward record books. Patients with incomplete or unavailable data in the medical archives were excluded.

### Institutional Review Board approval

Ethical approval was received from the Institutional Review Board at each institution. Both the University of Virginia IRB (HSR230211) and the University of Rwanda Ethics Committee approved the study, waiving the requirement for written informed consent.

### Clinical and demographic data collection

Patient identification numbers obtained from the hospital’s operative logbooks were used to obtain medical record files from the hospital archives. The CHUK Research Office and the Archives department granted access to medical records. The patient’s full name and date of birth were used as additional identifiers to ensure data was obtained from the correct paper chart. We collected data including patient age, sex, socio-demographic characteristics, comorbid conditions, ARM fistula classification(s), preoperative diagnostic workup, type of anorectoplasty completed, and postoperative outcomes. We categorized anorectal repair based on the number of stages completed to achieve a definitive repair. We considered colostomy creation, anorectoplasty, and colostomy closure as options. We identified patients who completed one-stage repair as those who had initial surgery as anorectoplasty. Patients who underwent a two-stage repair either had colostomy creation followed by anorectoplasty and concurrent colostomy closure (two-stage A) or colostomy creation and anorectoplasty, followed by a return for colostomy closure (two-stage B). Patients who underwent colostomy creation, anorectoplasty, and colostomy closure in three separate operations were considered to have undergone three-stage repair. The primary outcome was post-operative 30-day mortality. This was determined by an ongoing hospital stay of 30 days or more from surgery at CHUK, or a record of a follow-up visit more than 30 days after surgery at CHUK.

### Data Analysis

Demographic and clinical factors were reported for the entire cohort, as well as stratified by 30-day mortality status (yes/no) among patients with known 30-day survival status. Continuous variables were summarized using the mean with standard deviation (SD) or the median with interquartile range (IQR), and compared between groups using either the t-test or the Wilcoxon Rank Sum test, as appropriate. Categorical variables were presented as counts and proportions, and compared between groups using the Chi-square test or Fisher’s exact test, as applicable. Missing data was documented. Univariable logistic regression models were used to assess associations between patient characteristics and 30-day mortality. Results are reported as odds ratios (ORs) with corresponding 95% confidence intervals (CIs) and p-values. Due to the limited sample size, multivariable analysis was not performed. Statistical significance was defined as a two-sided p-value < 0.05. All statistical analyses were conducted using R version 4.3.2.

## Results

We identified 237 unique patient identifiers for infants who presented to CHUK with a diagnosis of ARM from January 2015 to December 2023. Medical records for 64 of 237 patients identified were readily available in medical archives for data collection. Two patients were excluded from data collection due to incomplete documentation. Our final study population included 62 patients. The demographic and baseline clinical characteristics of patients with ARM are shown in Table 1. Our study population included 37 male (59.7%) and 25 female (40.3%) patients. Patients had an average gestational age of 40 weeks and a mean birth weight of 2,813.8 kg (SD 68.3). We found that 25/30 patients (83.3%) were born in a healthcare facility. An ARM was diagnosed in 32 patients (94.1%) during the newborn exam, with two patients (5.9%) diagnosed after their first day of life. The median age at arrival at CHUK was 5 days (IQR 2, 208).

For the 62 patients with ARM, the type of ARM varied based on sex. For male patients with ARM, 20 (54.1%) had ARM without an associated fistula, three (8.1%) had a recto-bladder neck fistula, nine (24.3%) had a perineal fistula, two (5.4%) had a recto-urethral-bulbar fistula, and three (8.1%) had a recto-urethral-prostatic fistula. For female patients with ARM, 1 (4%) had ARM without an associated fistula, one (4%) had a recto-bladder neck fistula, two (8%) had a perineal fistula, 20 (80%) had a rectovestibular fistula, with some recorded as rectovaginal fistula, and 1 (4%) had a cloaca.

Thirteen patients (21.0%) had only a colostomy performed without a record of definitive repair. These patients were excluded from our analysis because they did not undergo definitive repair. The average age at the time of colostomy was 5 days. The average time between admission to CHUK and colostomy placement was 1 day, with an average length of stay after colostomy of 7 days. Three patients presented to CHUK with a colostomy that had already been completed at the referring hospital. We further evaluated the approach to definitive repair at CHUK. Fourteen patients (22.6%) underwent a one-stage repair with definitive anorectoplasty, one patient (1.6%) underwent a two-stage repair, and 27 patients (43.5%) underwent a three-stage repair. No two-stage B approach to repair was completed in this cohort. The most common anorectoplasty approach in our cohort was the posterior sagittal anorectoplasty (PSARP), which was completed in 40 (64.5%) patients. Our analysis of staged repair excluded seven patients because there were no records of complete repair, either due to mortality or loss to follow-up. The average age of patients who had completed definitive repair was 263 days (IQR 184, 499). The average length of stay after definitive repair was 5 days (IQR 3, 7). The average time between colostomy and anorectoplasty was 257 days (IQR 193, 331).

Post-operative 30-day follow-up was missing in 23 patients. Among the 39 patients with known 30-day status, seven patients (18%) died within 30 days of the initial procedure at CHUK. Twenty-four patients (52.2%) had an additional congenital anomaly: two with atrial septal defects (3.2%), five with ventricular septal defects (8.1%), three with trisomy 21 (4.8%), and 9 with a VACTERL association (14.5%). Of those who died within 30 days, 100% of the deaths occurred within 7 days of the initial surgery at CHUK. Table 3 shows the demographic and clinical characteristics associated with 30-day mortality. The number of records varied for each characteristic. In the mortality cohort, the median birth weight was 2300g (SD, 462), compared to a median birth weight of 2877g (SD, 516) among those who survived beyond 30 days (p = 0.018). Additionally, weight at the initial surgery, colostomy, or anorectoplasty was negatively associated with 30-day mortality. Therefore, low weight (<3000g) at surgery was significantly associated with a 94% higher risk of mortality. And, although there was a trend towards a higher mortality within 30 days in infants with additional congenital anomalies (83.3% vs 54.5%), this was not statistically significant (p = 0.36).

Table 4 presents the univariable logistic regression and odds ratios for the predictors of mortality within 30 days of an ARM-associated operation at CHUK. We found that the presence of any fistula, compared with isolated ARM without an associated fistula and a weight less than 3000g at initial surgery, was predictive of mortality within 30 days. For patients who had any fistula, the odds of mortality with a fistula were 93% lower than in patients without a fistula. For patients who had an initial weight at surgery greater than 3000g, the odds of mortality were 94% lower than in patients who had weighed less than 3000g. Multivariable analysis was not conducted due to the limited sample size.

Overall, 35 patients (56%) experienced a complication in the postoperative period. The most common postoperative complications were sepsis in 7 patients (11.3%) and colostomy prolapse in 7 patients (11.3%). Five patients (8%) had anal stricture or stenosis, four patients (6.5%) had surgical site dehiscence, four patients (6.5%) had a surgical site infection, and four patients (6.5%) had stoma obstruction/stenosis. Two patients required anorectoplasty revision (3.2%), and two patients had postoperative fever (3.2%).

## Discussion

Anorectal malformations, often referred to as imperforate anus, encompass a wide range of congenital malformations that can affect the rectum and genitourinary tract, with a reported overall mortality between 1.4% and 30%. A three-stage approach, which includes colostomy creation, definitive anorectoplasty, and colostomy closure, is the most common method for repair and management of this condition worldwide.^21^ The presence of other congenital anomalies, most commonly involving the urogenital, cardiovascular, vertebral, and nervous systems, is often associated with higher mortality in neonates compared to those with isolated ARM.^22^

This study presents the first attempt to investigate the management of and surgical outcomes for patients with ARM at CHUK in Rwanda by fellowship-trained pediatric specialists. A prior study describing anorectal malformations in Rwanda was conducted by Makanga et al, who reviewed 648 pediatric surgery cases for gastrointestinal conditions between 2002 and 2007 at the Teaching Hospital of Butare, Rwanda, of which 46 (7%) were for anorectal malformations.^23^ Although our study included 62 patients, we identified over 200 patients treated at a single tertiary referral hospital in Rwanda over 8 years, demonstrating that these conditions are frequently encountered in hospitals throughout the country.

At least 50% of patients arrived at CHUK at more than 5 days old, demonstrating some element of delay in management. Other studies of anorectal malformations conducted in LMICs have identified delays in diagnosis as a significant barrier to care. In Uganda, Oyania et al. conducted a study on low ARMs. They found that only 26% of patients were diagnosed within 24 hours of birth, while 80% of cases were diagnosed by parents/caregivers.^24^ Similarly, a retrospective review by Adejuiyigbe et al in South-West Nigeria revealed that 86% of patients presented after 24 hours of life, with signs of obstruction in 25%.^25^ When a newborn exam was reported in the medical record, there was a 94% detection rate. The limitations of the medical records limited our ability to understand the overall detection rate at the time of birth. This rate of detection also exceeds that of many HICs.^26^ Delays in presentation of patients with ARM have been reported to lead to progression of neonatal intestinal obstruction, sepsis, aspiration pneumonia, intestinal perforation, and, in some instances, death.^27^

Geographic variation in types of anorectal malformations seen contributes to surgeons’ expertise and, consequently, care outcomes. In our study population, the most common ARM in males was an ARM without a fistula, as seen in Kenya; however, in Ethiopia, Malawi, ARM with rectourethral fistula has been more commonly reported in males.^27,28^ In females, rectovestibular fistula (40%) and rectovaginal fistula (40%) were the most frequently observed types of anorectal malformation. On confirmation with the operating surgeon, the observed prevalence of rectovaginal fistula is likely due to a documentation error and more likely represents rectovestibular fistula instead. The three-stage repair was the most commonly implemented approach for patients in our population, and the most common approach to anorectoplasty was posterior sagittal anorectoplasty (PSARP). The availability of medical archives of only 62 patients among the 237 patients initially identified limited our ability to fully characterize the existing distribution of types of ARMs treated at CHUK.

Overall mortality in our study was 17.9% while postoperative morbidity was 56% for the patients with available data. All deaths occurred within 7 days of initial surgery. Sepsis was one of the most common postoperative complications, representing 20% of postoperative complications recorded. Among the seven patients who died, 3 (43%) developed sepsis in the postoperative period. Makanga et al reported a mortality rate of 13% in their earlier study of ARMs in Rwanda, which is lower than our study, and postoperative complications in 14 cases (30%), with 10 of those being cases of infection. Similar findings were also described by Chongera et al in Tanzania, who reported a postoperative complication rate of 17.2% and a mortality rate of only 9.9%.^29^ Mortality data were missing for 37% of our study population; therefore, we may have overestimated mortality.

Significant drivers of mortality identified in our study were a weight of less than 3000g at initial surgery and an isolated ARM without fistula in our cohort. We did not find an association between congenital heart defects and mortality in our cohort; however, such a relationship has been previously described in other studies, though mortality was increased in our group with an associated anomaly.^17^ An associated congenital anomaly has also been shown to be more associated with poor outcomes when compared to the presence or lack of a fistula.^30^ An unexpected finding in our study was the time between colostomy and anorectoplasty. Our study was not designed to evaluate the provision of support and supplies for maintaining colostomies after colostomy creation. However, the socioeconomic and quality of life effects due to colostomies are well documented in the literature; thus, we expect that there is a socioeconomic burden associated with the maintenance of colostomy.^31,32^ Muzira et al conducted a qualitative study in Uganda for caregivers of pediatric patients who had received colostomies for ARMs. They found that 93% of caregivers suffered job losses due to the burden of stoma care.^19^

There are several potential limitations to our study. Our sample size included only 62 patients, although we identified over twice this number of patients who had presented with ARMs. Despite identifying over 200 patients, a significant number of patient records were inaccessible from the central archives office located on the CHUK campus. Another limitation was the quality of the available data. There was also variation in documentation such that patients’ records did not capture all relevant demographic and baseline characteristics. As CHUK is a referral hospital, patients presented from a wide variety of health centers, which did not all capture the same demographic and clinical characteristics for each patient. The medical records were also mainly handwritten. Abstracting data from handwritten records was sometimes a challenge due to poor legibility. It included acronyms or shorthand that may have had a context unfamiliar to our study team, both of which could have increased the chances of reporting errors.

Additionally, natural degradation of paper made some pages of documentation from paper files illegible. There was also missing information in the paper records in the archive, potentially the result of staff turnover and differing documentation styles used between the operative logbook, forms in the medical records folders, and folder organization by medical records staff. Potential targeted quality improvement initiatives could be aimed at standardizing the reporting of newborn examinations across the tiered health systems that already exist in Rwanda.

Our study covers a significant time period that overlaps with the introduction of fellowship-trained pediatric surgeons in Rwanda. Prior to this, surgeries in pediatric patients were done by general surgeons with the intermittent availability of pediatric surgeons in the country. A study assessing the need for pediatric surgery capacity in Rwanda by Petroze et al estimated that over 50% of surgical need in Rwanda is in pediatric cases, and a sample survey of the population demonstrated surgically treatable conditions in 7% of children, highlighting the need for fellowship-trained pediatric surgeons.^33^ With the presence of one pediatric surgeon in the country, Rwanda’s pediatric surgery workforce density (PSWD) used to sit at 0.020 per 100,000, which was below the critical threshold of 0.37 – 0.4 per 100,000 associated with improved surgical outcomes and improved pediatric survival.^9^ This capacity has improved over the last decade with a current pediatric surgery fellowship training program in Rwanda. Referrals to tertiary centers, such as CHUK, for pediatric surgical attention can contribute to delays in treatment. In response to this gap in access to surgical care, Rwanda has implemented a National Surgical, Obstetric, and Anesthesia plan to increase access to surgical care and address this challenge.^34^ Additional quality improvement initiatives with the focus of care bundles for patients < 3000g could also help improve outcomes.

## Conclusion

Anorectal malformations are among the most frequent congenital anomalies of the gastrointestinal system. The complex nature of these malformations also necessitates access to specialized pediatric care. Morbidity and survival rates depend on the specific type of malformation and the severity of any associated comorbidities. While the mortality rate in this cohort was higher than in high-income countries (HICs), it was lower than typically reported in low- and middle-income countries (LMICs). This improvement may be due to the enhanced care offered by fellowship-trained pediatric specialists. Additional efforts to improve outcomes include standardizing medical documentation across the existing tiered health system and care bundles for specific groups.

## Figures and Tables

**Figure 1:**
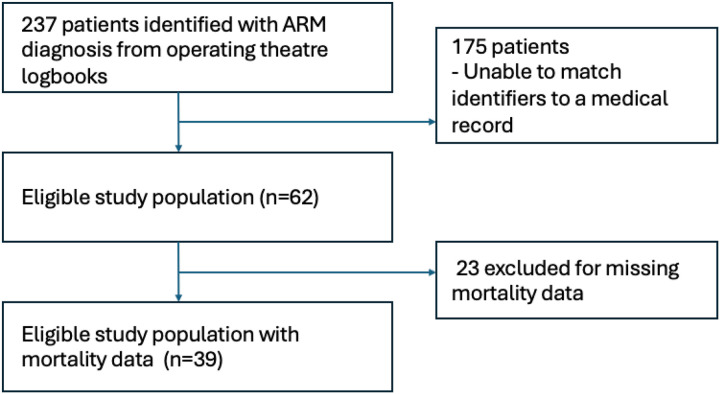
Flowchart demonstrating the definition of the study population and reasons for exclusion from our analysis.

**Table 1:** Patient Demographics and Baseline Clinical Characteristics (n = 62).

Birth weight in grams, mean (SD)		2813.8 (683.5)
Gestational age in weeks (median [IQR])		40 (40, 40)
Sex		
Male, n (%)		37 (59.7%)
Female, n (%)		25 (40.3%)
Age at arrival to CHUK (days), median (IQR)		5 (2, 208)
Health insurance		
Yes, n (%)		60 (96.8)
No, n (%)		2 (3.2)
Birth Location		
Hospital/Health Center, n (%)		25 (83.3)
Other, n (%)		5 (16.7)
ARM diagnosed on newborn exam when newborn exam was available		
Yes, n (%)		32 (94.1)
No, n (%)		2 (5.9)
Type of Anorectal Malformation divided by sex		
Male, n (%)	ARM without a fistula	20 (54.1)
	Perineal Fistula	9 (24.3)
	Recto-bladder neck Fistula	3 (8.1)
	Recto-urethral bulbar Fistula	2 (5.4)
	Recto-urethral prostatic Fistula	3 (8.1)
Female, n (%)	ARM without a fistula	1 (4)
	Recto-bladder neck Fistula	1 (4)
	Rectoperineal Fistula	2 (8)
	Rectovestibular Fistula	10 (40)
	Rectovaginal Fistula***	10(40)
	Cloaca	1 (4)
Additional congenital anomaly, n (%)		24 (52.2)

*** likely documentation error based on clarification by pediatric surgeon at CHUK.

**Table 2:** Clinical and Operative Characteristics (n = 62)

Colostomy only, n (%)	13 (21.0)
Multi-Stage ARM repair, n (%) *	
One-Stage	14 (22.6)
Two-Stage A	1 (1.6)
Two-Stage B	0 (0)
Three-Stage	27 (43.5)
Missing data	7 (11.3)
Age at colostomy in days, median (IQR)	5 (3, 151)
Age at anorectoplasty in days, median (IQR)	263 (184, 499)
Time from admission to colostomy at CHUK in days, median (IQR)	1 (1, 2)
Distal Colostogram completed prior to repair, n (%)↟	10 (17.5)
Type of definitive repair, n (%) ↥	
ASARP	4 (8.2)
Not specified anorectoplasty	4 (8.2)
PSARP	40 (81.6)
PSARVUP	1 (16)
Length of stay after colostomy in days, median (IQR)	7 (3, 9)
Length of stay after anorectoplasty in days, median (IQR)	5 (3, 7)
Time from colostomy to anorectoplasty in days, median (IQR)	257 (193, 331)
Discharged home with colostomy after anorectoplasty☒	40

* Excluded patients without a record of completed repair prior to arrival at CHUK. Two-stage A: colostomy creation followed by anorectoplasty and concurrent colostomy closure. Two-stage B: colostomy creation and anorectoplasty, followed by a return for colostomy closure

↟ 5 missing

↥ 13 missing

☒ 9 missing

**Abbreviations**: ASARP = anterior sagittal anorectoplasty; PSARP = posterior sagittal anorectoplasty; PSARVUP = posterior sagittal anorectal vaginal urethroplasty

**Table 3:** Demographic and Clinical Characteristics associated with 30-day mortality (n=39)

		Mortality		p-value
		Yes (n=7)	No (n=32)	
Sex, n (%)	Male	6 (85.7)	16 (50.0)	0.113
	Female	1 (14.3)	16 (50.0)	
Birth weight in grams, mean (SD) ↻		2300 (462)	2877 (516)	**0.018**
Distance travelled in kilometers, mean (SD) ↟				0.486
	<50	2 (28.6)	15 (48.4)	
	50–99	1 (14.3)	7 (22.6%)	
	>=100	4 (57.1)	9 (29.0)	
Age at Initial Surgery in days, mean (SD)↥				0.290
	<=7d	7 (100.0)	17 (56.7)	
	8–30d	0 (0.0)	4 (13.3)	
	31–365	0 (0.0)	6 (20.0)	
	>365	0 (0.0)	3 (10.0)	
Any Associated Fistula, n (%)⇑		1 (14.3)	23 (71.9)	**0.008**
	Rectovaginal (likely rectovestibular fistula)***	1 (14.3)	6 (18.8)	
	Rectovestibular	0 (0.0)	10 (31.2)	
	Perineal	0 (0.0)	2 (6.2)	
	Recto-urethral prostatic	0 (0.0)	2 (6.2)	
	Recto-urethral bulbar	0 (0.0)	2 (6.2)	
	Recto-bladder neck	0 (0.0)	2 (6.2)	
Weight at Initial Surgery in grams, mean (SD)				**0.011**
	<2500	6 (85.7)	4 (19.0)	
	2500–2999	0 (0.0)	0 (0.0)	
	=>3000	1 (14.3)	12 (57.1)	
Presence of Additional Congenital Anomaly, n (%) ☒		5 (83.3)	12 (54.5)	0.355
Initial Surgery was Colostomy, n (%)		7 (100.0	0 (0.0)	>0.999
Completed Colostogram prior to Surgery, n (%) ☒		0 (0.0)	8 (27.6)	0.299
Surgery Prior to Transfer, n (%)		0 (0.0)	3 (9.4)	>0.999

↟ 1 missing

↥ 2 missing

☒ 4 missing

☒ 11 missing

↻ 13 missing

**Table 4:** Univariable logistic regression for predictors of mortality (n=39)

		n (%)	OR (95% CI)	p-value
Sex	Female	17 (43.6)	0.17 (0.02,1.55)	0.115
	Male	22 (56.4)	1	
Birth weight in grams ↻				
	<2500	10 (38.5)	1	
	2500 – 2999	6 (23.1)	0.20 (0.02, 2.39)	0.203
	>=3000	10 (38.5)	0.11 (0.01, 1.24)	0.074
Distance travelled in kilometers (km) ↟				
	<50	17 (44.7)	1	
	50–99	8 (21.1)	1.07 (0.08,13.90)	0.958
	>=100	13 (34.2)	3.3 (0.50, 22.02)	0.211
Age at Initial Surgery in days, median (IQR)↥		5 [3, 18]	0.92 (0.70, 1.20)	0.526
ARM features				
No associated fistula		15 (38.5)	1	
Any type of associated fistula		24 (61.5)	0.07 (0.01, 0.62)	**0.018**
Weight at Initial Surgery in grams				
	<2500	10 (35.7)	1	
	2500–2999	5 (7.9)	0.00 (0.00,Inf)	0.995
	=>3000	13 (46.4)	0.06 (0.01,0.61)	**0.018**
Presence of Additional Congenital Anomaly ☒		17 (60.7)	4.17 (0.42,41.76)	0.225
Completed Colostogram prior to Surgery ☒		8 (22.9)	0.00 (0.00,Inf)	0.994
Surgery Prior to Transfer		3 (7.7)	0.00 (0.00,Inf)	0.994

↟ 1 missing

↥ 2 missing

☒ 4 missing

☒ 11 missing

↻ 13 missing

**Table 5:** Postoperative Complications (n=62)

	n (%)
Anorectoplasty revision	2 (3.2%)
Sepsis	7 (11.3%)
Surgical Site Dehiscence	4 (6.5%)
Surgical Site Infection	4 (6.5%)
Anal Stricture or Stenosis	5 (8.0%)
Colostomy prolapse	7 (11.3%)
Stoma obstruction/stenosis	4 (6.5%)
Fever	2 (3.2%)

## Data Availability

De-identified datasets used and analyzed in this study are available from the corresponding author upon reasonable request.
